# Cyclic Compressive Stress Regulates Apoptosis in Rat Osteoblasts: Involvement of PI3K/Akt and JNK MAPK Signaling Pathways

**DOI:** 10.1371/journal.pone.0165845

**Published:** 2016-11-02

**Authors:** Fanglong Song, Yi Wang, Dawei Jiang, Tianchen Wang, Yinquan Zhang, Hui Ma, Yifan Kang

**Affiliations:** Department of Orthopedics, Third Affiliated Hospital of PLA Second Military Medical University, Shanghai, 200433, China; Duke University School of Medicine, UNITED STATES

## Abstract

It is widely accepted that physiological mechanical stimulation suppresses apoptosis and induces synthesis of extracellular matrix by osteoblasts; however, the effect of stress overloading on osteoblasts has not been fully illustrated. In the present study, we investigated the effect of cyclic compressive stress on rat osteoblasts apoptosis, using a novel liquid drop method to generate mechanical stress on osteoblast monolayers. After treatment with different levels of mechanical stress, apoptosis of osteoblasts and activations of mitogen-activated protein kinases (MAPKs) and PI3-kinase (PI3K)/Akt signaling pathways were investigated. Osteoblasts apoptosis was observed after treated with specific inhibitors prior to mechanical stimulation. Protein levels of Bax/Bcl-2/caspase-3 signaling were determined using western blot with or without inhibitors of PI3K/Akt and phosphorylation of c-jun N-terminal kinase (JNK) MAPK. Results showed that mechanical stimulation led to osteoblasts apoptosis in a dose-dependent manner and a remarkable activation of MAPKs and PI3K/Akt signaling pathways. Activation of PI3K/Akt protected against apoptosis, whereas JNK MAPK increased apoptosis via regulation of Bax/Bcl-2/caspase-3 activation. In summary, the PI3K/Akt and JNK MAPK signaling pathways played opposing roles in osteoblasts apoptosis, resulting in inhibition of apoptosis upon small-magnitude stress and increased apoptosis upon large-magnitude stress.

## Introduction

It is well known that mechanical stress plays an important part in bone metabolism. It is also firmly established that mechanical loading of bone results in increased bone formation and remodeling[1, 2]. However, when physiological mechanical stimulation is absent, for example, during exposure to an environment of microgravity, after prolonged bed rest or following joint immobilization after surgery, bone resorption increases and bone mass is lost[3, 4]. Mechanical loading of bone in vivo causes tissue deformation and results in the application of mechanical stimulation to cells embedded in the bone matrix, and the activity of bone cells is regulated in response to the changes in mechanical environments[1, 5]. In order to investigate the mechanical response of cells, a variety of methods have been employed to simulate the stress environment of osteocytes and osteoblasts in the mineralized matrix of bone, including fluid shear stress, cyclic stretch, continuous compressive force and mechanical stress generated by liquid perfusion or compressed air[6–10]. However, the response of monolayer osteoblasts to mechanical stress generated by liquid drops has never been reported. It is widely accepted that physiological mechanical loading leads to an anti-apoptotic effect and increased proliferation and differentiation of osteoblasts which results in extracellular matrix formation[2, 6, 11–13]. At present, some studies have suggested that mechanical overloading acts as a negative regulator of bone formation and induces cell apoptosis, but the precise cellular mechanism is poorly understood[7, 14–16].

Apoptosis, or programmed cell death, is a physiological process leading to elimination of unwanted cells within living tissues, which is essential in the regulation of tissue turnover in long-lived mammals[17]. Apoptosis of osteoblasts is a significant event in bone, as approximately 70% of osteoblasts are thought to undergo apoptosis in the process of bone remodeling[18]. In bone tissue, regulation of osteoblast apoptosis is thought to play a key role in the maintenance of healthy bone and skeletal architectural integrity[19–21].

Extracellular stimuli, such as mechanical stimuli, growth factors, and oxidative stress, activate key intracellular signaling pathways, in particular, PI3-kinase (PI3K)/Akt and mitogen-activated protein kinases (MAPKs), to stimulate cytoplasmic and nuclear effectors which regulate various cellular functions involving cell growth, differentiation, cytokine production and apoptosis[22–25]. It has been determined that the effect of mechanical stress is mediated by these two signaling pathways[6, 26–28]. Although they belong to the same family of intracellular signaling regulators, the three major MAPKs, which include extracellular signal-regulated kinase p44/42 MAPK (ERK1/2), p38 MAPK (p38) and c-Jun N-terminal kinase (JNK), play different roles in cells in response to mechanical stimulation, and their effects on mechanical stress-induced apoptosis are still controversial[10, 14, 15]. It has been shown that ERK activated by moderate mechanical stretch contributes to differentiation of osteoblasts and does not affect apoptosis[15], while other studies have reported that ERK inhibits apoptosis induced by cyclic stretch in osteoblasts[14]. In addition, it has been demonstrated that ERK contributes to cell apoptosis induced by static mechanical stress[10]. JNK activated by large-magnitude mechanical stretch not only suppresses differentiation but also leads to cell apoptosis[15]. Finally, p38 that is activated by large-magnitude mechanical stretch induces local recruitment of pre-osteoclasts and subsequent osteoclastogenesis; however, it may also lead to apoptosis when activated by static mechanical stress[10, 15].

The growth of cells is also regulated through the PI3K/Akt pathway[29]. It has been reported that inhibition of the PI3K/Akt pathway can induce cell death[30]. Phosphorylated Akt functions as a survival signal partially by inactivating two pro-apoptotic proteins, Bad and caspase-9[31]. Studies using MC3T3-E1 osteoblasts suggest that mechanical stimulation such as fluid shear stress serves as a signal to inhibit apoptosis through activation of the PI3K/Akt pathway[1]. However, the activation of the PI3K/Akt pathway by different levels of mechanical stress has not been well documented, and the anti-apoptotic effect may change as the levels of stress change.

In this study, we used a device to simulate the stress environment of osteoblasts by applying mechanical loading generated by water drops on monolayer cells to test the hypothesis that the apoptotic effect in osteoblasts is regulated by different levels of mechanical stress. To investigate the mechanism underlying mechanical stress regulation of apoptosis in osteoblasts, we tested the activation of MAPKs and PI3K/Akt signaling pathways and demonstrated that mechanical stimulation regulated osteoblast apoptosis by activating the MAPKs and PI3K/Akt signaling pathways at different levels of stress. In particular, we observed that JNK and Akt had opposing effects on apoptosis by regulating Bax/Bcl-2/caspase-3 activation, which resulted in inhibition of apoptosis upon small-magnitude stress and increased apoptosis upon large-magnitude stress. This study thus provides preliminary findings on the regulatory mechanism of osteoblast apoptosis under mechanical stress and offers a new method to simulate the stress environment of osteoblasts.

## Materials and Methods

### Antibodies and reagents

Antibodies against phospho-ERK(Thr202/Tyr204) MAPK, phospho-SAPK/JNK(Thr183/Tyr185), phospho-p38(Thr180/Tyr182) MAPK, ERK MAPK, SAPK/JNK, p38 MAPK, phospho-Akt(Ser473), Akt, Bad, phospho-Bad(Ser136), Bax, Bcl-2, β-Actin (13E5), Caspase-3, Cleaved caspase-3 and the appropriate anti-rabbit horseradish peroxidase (HRP)-conjugated secondary antibody were purchased from Cell Signaling Technology, USA. Reagent Sources including PD98059 (ERK MAPK inhibitor), SP600125 (JNK inhibitor), SB203580 (p38 MAPK inhibitor) and LY294002 (PI3K/Akt inhibitor) were purchased from Sigma, USA. The enhanced chemiluminescence (ECL) detection substrate was purchased from Thermo Fisher Scientific, USA.

### Cell culture and mechanical stress application

**Fig 1:**
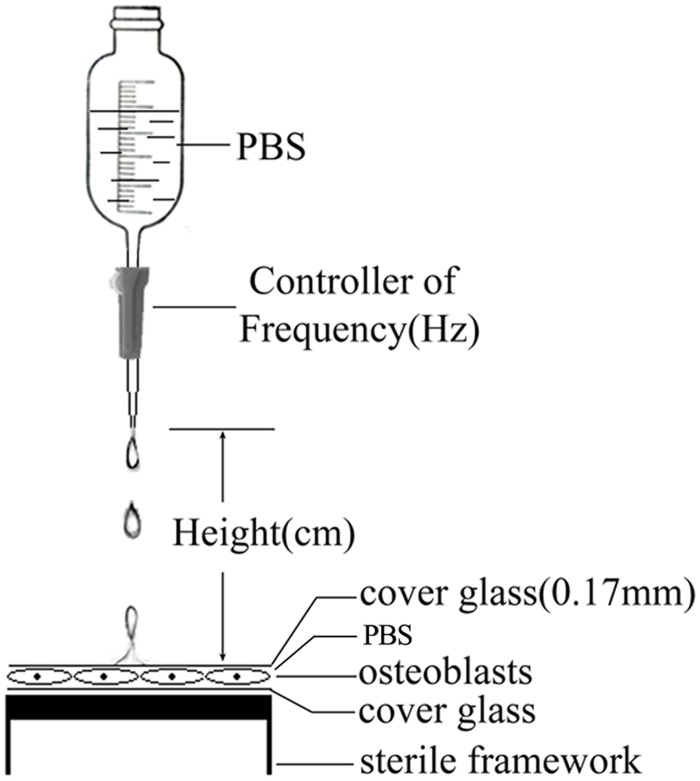
Diagrammatic representation of the device used for cyclic compressive stress.

Osteoblasts were isolated from the calvaries from neonatal rats (7 days) under a protocol approved by the animal research committee of PLA Second Military Medical University. Calvaries were isolated aseptically and minced and digested with 0.25% trypsin for 30 minutes at 37°C. The slices were then digested with 0.2% collagenase II at 37°C for 4 hours. Cells released were pooled and cultured in dulbecco's modified eagle medium/f12 (DMEM/F12) supplemented with 10% fetal bovine serum (FBS) and 1% penicillin-streptomycin in a humidified atmosphere incubator containing 95% air and 5% CO_2_ at 37°C with medium changed every other day. Cells were passaged when they were 90–95% confluent by trypsinization and seeded at a density of approximately 10^4^ cells/cm^2^ on round glasses (φ = 25mm) covered with poly-lysine. Cells on glasses were exposed to cyclic mechanical stress when they were 90–95% confluent at a density of approximately 1×10^5^ cells /cm^2^ and the number of cells on each glass was about 5×10^5^(Fig 1).

Briefly, thin cover glass plates were placed over the confluent cell layers on the glasses. The cyclic mechanical stress was adjusted by drops of PBS to the glass plates. Cells were subjected to different frequency (Hz) and height (cm) of mechanical stress for 30min. The frequency was calculated using the following equation:

$$frequency=\frac{1}{t}$$

“t” represented time span between two drops(s). Control cells were covered with a thin glass plate without any liquid dropping on it. The experiment of mechanical stress application was performed in a humidified atmosphere incubator containing 95% air and 5% CO_2_ at 37°C in which the temperature, humidity, oxygen content and pH were controlled strictly.

### TUNEL staining

After treatment of mechanical stress, TUNEL staining was performed using an in situ cell death detection kit (Roche, Switzerland) according to the manufacturer’s instructions. Briefly, cells were fixed with 4% (v/v) paraformaldehyde for 1 h at room temperature and then permeabilized with 0.1% (v/v) Triton X-100 for 2 minutes on ice. Washed twice in PBS, the cells were then incubated with the TUNEL reaction mixture for 1 h at 37°C. After washed with PBS, cells were stained with DAPI (4', 6-diamidino-2-phenylindole) for 5 minutes and fluorescent images were acquired under a fluorescence microscope. Evaluations of cell apoptosis were performed independently by two observers in a blinded fashion. A minimum of 6 fields were randomly selected and TUNEL-positive cells (cells with DNA fragmentation and condensed nuclei) were counted in each field. Representative images matching the conclusions were presented. The percentage of positive cells was calculated by (number of positive cells/total number of cells)×100%.

### Caspase-3 activity assay

The activity of caspase-3 was determined by cleavage of chromogenic caspase substrates, the chromophore DEVD-p-nitroaniline (pNA), which is a caspase-3 substrate using the caspase-3 activity assay kit (Beyotime, Shanghai, China). Briefly, after treatment of mechanical stress, cells were lysed by using lysis buffer for 15 min on ice, and centrifuged at a speed of 18,000rpm for 15 min at 4°C. Each of the protein samples was then added to the reaction buffer containing DEVD-pNA according to the manufacturer’s instructions and the mixture was incubated at 37°C for 2 h. The absorbance of pNA was detected using a microplate spectrophotometer (Biotek, USA) at 405 nm. Caspase-3 activity was quantified as the fold of enzymatic activity in apoptotic samples compared to that of control samples.

### Flow cytometric analysis

Cell apoptosis was analyzed using Annexin V apoptosis detection kit (Beyotime, Shanghai, China) by flow cytometry according to the manufacturer’s protocol. Briefly, cells were harvested after mechanical stress application and washed three times with PBS and resuspended in binding buffer. Then, cells were incubated in Annexin-V/PI at room temperature for 15 min in the dark and analyzed by flow cytometry.

### Inhibitor treatment

For inhibitor studies, rat osteoblats were pre-incubated with 10μM PD98059 (ERK MAPK inhibitor), 10μM SP600125 (JNK inhibitor), 10μM SB203580 (p38 MAPK inhibitor) or 10μM LY294002 (PI3K/Akt inhibitor) in DMSO for 1 h followed by application of cyclic mechanical stress. Cells supplemented with DMSO (vehicle) without inhibitor were used as controls.

### Western blot and data quantification

After treatment of mechanical stress, cells were washed with cold PBS and lysed in a lysis buffer (Beyotime, Shanghai, China). Lysates were mixed and incubated on ice for 15 min and then cell debris was spun down at a speed of 14,000rpm for 15 min at 4°C. Concentrations of proteins samples in supernatant were determined using the bicinchoninic acid (BCA) method with the protein assay kit(Beyotime, Shanghai, China). Equal quantities (20μg) of proteins samples (dissolved in 5× loading buffer) were separated using SDS-PAGE gels (5% stacking gel and a 10% running gel) and then electro-transferred to polyvinylidene fluoride (PVDF) membranes by wet transfer method at 250mA for 30min. After blocking in 5% bovineserum albumin (BSA) in TBS with 0.1% Tween-20(TBST) at room temperature for 1 h, membranes were then incubated with primary antibodies(1:1000 except for ERK(1:2000) and β-actin(1:2000)) overnight at 4°C. After washing with TBST, blots were then incubated with HRP-conjugated secondary antibodies (1:2000) at room temperature for 1 h. Protein bands were visualized using ECL reagents. The intensity values of each phosphorylated kinase were quantified using densitometric analysis with ImageJ 1.36 and normalized to the intensity of corresponding total protein bands. Unless otherwise stated, β-actin was used as an internal control.

### Statistical analysis

All results were presented as the mean±standard deviation (SD). Statistical significance was determined using SPSS 13.0 for Windows by one-way analysis of variance (ANOVA) followed by multiple comparisons performed with post hoc Bonferroni test. *P* < 0.05 was considered statistically significant. All experiments were repeated at least three times.

## Results

### Mechanical stress regulates apoptosis and activation of caspase-3 in osteoblasts

**Fig 2:**
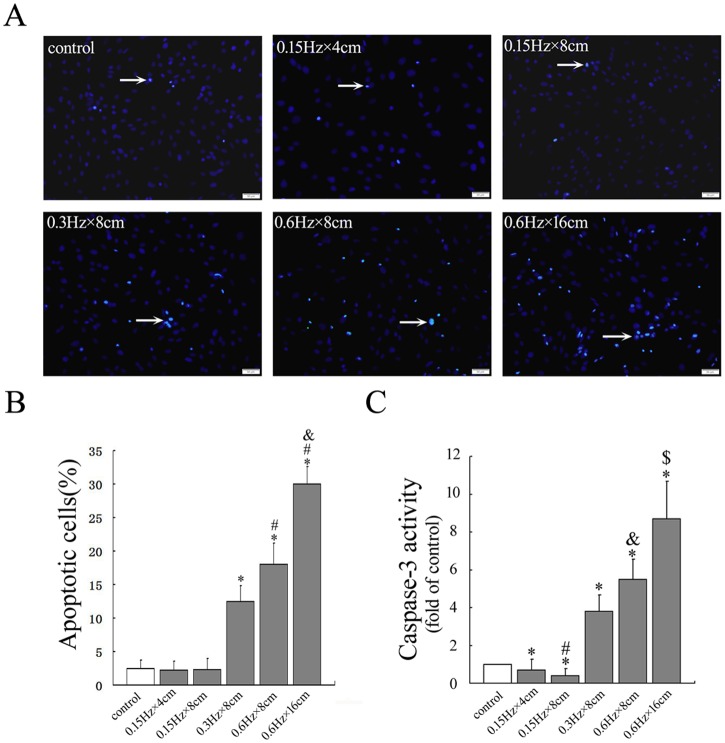
Effect of different levels of cyclic compressive stress on apoptosis in osteoblasts. Osteoblasts were treated with different levels of mechanical stress (0.15 Hz×4cm,0.15Hz×8cm, 0.3Hz×8cm, 0.6Hz×8cm, 0.6Hz×16cm)(0 as control) for 30min and then cultured for another 24h. Cells were harvested and measured for apoptosis and caspase-3 activity. A. The apoptotic cells were determined by TUNEL staining. Scale bar = 50μm. B. Quantitative analysis of apoptotic cells in each group. *P<0.05 versus control group; #*P*<0.05 versus 0.3Hz×8cm group; &*P*<0.05 versus 0.6Hz×8cm group. C. Quantitative analysis of caspase-3 activity in each group. *P<0.05 versus control group; #*P*<0.05 versus 0.15Hz×4cm group; &*P*<0.05 versus 0.3Hz×8cm group; $*P*<0.05 versus 0.6Hz×8cm group. Each value is presented as mean ± SD of three independent experiments and data of the treatment group was expressed as fold change vs. that of control group (labeled as “1.00”).

To determine the effects of mechanical stress on osteoblasts apoptosis, cells were treated with different levels of mechanical stress(0.15 Hz×4cm,0.15Hz×8cm, 0.3Hz×8cm, 0.6Hz×8cm, 0.6Hz×16cm)(0 as control) for 30min and then cultured for another 24h and apoptosis was assessed using TUNEL staining and caspase-3 activity assay. Small-magnitude stress of 0.15 Hz×4cm and 0.15Hz×8cm decreased osteoblasts apoptosis compared with that of control. Large-magnitude stress of 0.3Hz×8cm, 0.6Hz×8cm, 0.6Hz×16cm promoted apoptosis in a dose dependent manner (*P* < 0.05) after mechanical stimulation compared with the control cells. The results of caspase-3 activity assay revealed the same trend. However, small-magnitude stress of 0.15 Hz×4cm and 0.15Hz×8cm decreased caspase-3 activity in osteoblasts significantly (*P* < 0.05) compared with that of control, as shown in Fig 2. Therefore, the cells were treated with 0.15 Hz×4cm, 0.15Hz×8cm and 0.6Hz×8cm for duration of 30min in the subsequent experiments.

### MAPKs and PI3K/Akt were activated by different levels of mechanical stress

**Fig 3:**
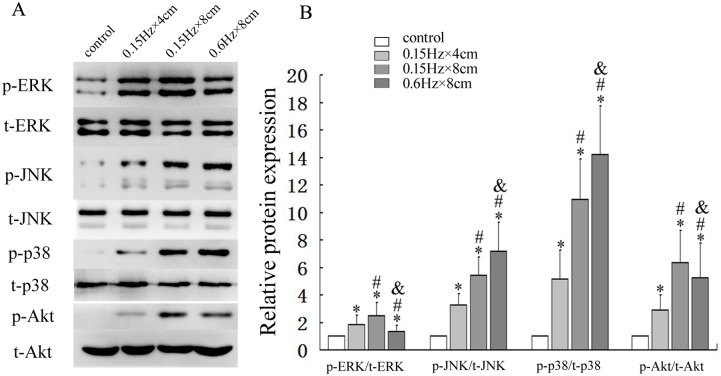
Activation of MAPKs and Akt in response to different levels of mechanical stress. Osteoblasts were stimulated with different levels of mechanical stress for 30 min. The kinase activity of MAPKs and PI3K/Akt were detected by western bolt using p-ERK, p-JNK, p-p38 and p-Akt antibodies. (A) The expression levels of MAPKs and Akt examined by western blot. (B) Quantitative analysis of activations of MAPKs and Akt. *P<0.05 versus control group; #*P*<0.05 versus 0.15Hz×4cm group; &*P*<0.05 versus 0.15Hz×8cm group. Each value is presented as mean ± SD of three independent experiments and data of the treatment group was expressed as fold change vs. that of control group (labeled as “1.00”).

To explore the roles of MAPK and PI3K/Akt pathways in the mechanical stress response, cells were treated with different levels of mechanical stress (0.15 Hz×4cm,0.15Hz×8cm, 0.6Hz×8cm)(0 as control) for 30min and the effect of mechanical stimulation on the phosphoralation of ERK, JNK, p38 and Akt were analyzed. Upon mechanical stress loading, ERK and Akt were strongly activated by small-magnitude stresses but were inhibited by large-magnitude stress respectively (*P* < 0.05). On the other hand, as the levels of stress increased, phosphorylation of p38 and JNK increased in a dose-dependent manner (*P* < 0.05) (Fig 3).

### JNK MAPK and PI3K/Akt pathways were required in the regulation of osteoblast apoptosis

**Fig 4:**
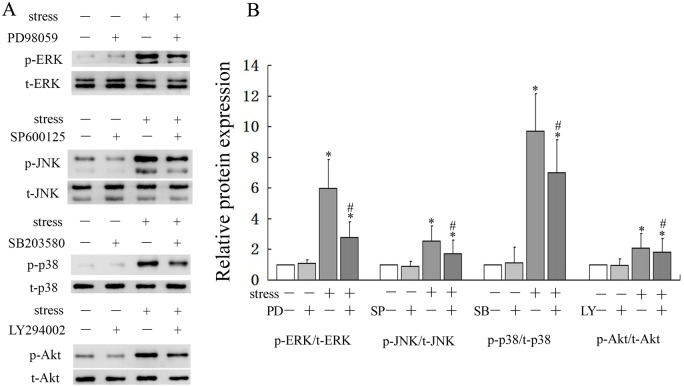
Effect of mechanical stress and specific inhibitors on phosphorylation of MAPKs and Akt. Osteoblasts were treated with mechanical stress of 0.6Hz×8cm(0 as control) for 30min in the presence or absence of SP600125, SB203580, PD98059 and LY294002. (A) Western blot analysis of the protein levels of p-ERK, p-JNK, p-p38 and p-Akt. (B) Quantitative analysis of the ratio of p-ERK/t-ERK, p-JNK /t-JNK, p-p38/t-p38 and p-Akt/t-Akt. *P<0.05 versus control group; #*P*<0.05 versus loaded group. Each value is presented as mean ± SD of three independent experiments and data of the treatment group was expressed as fold change vs. that of control group (labeled as “1.00”).

**Fig 5:**
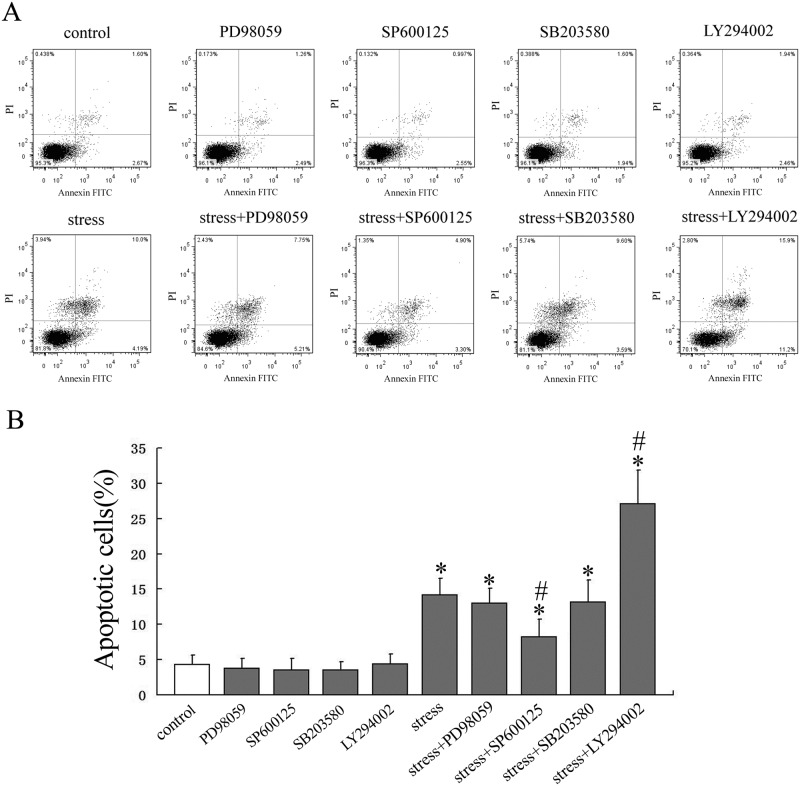
Effect of large-magnitude mechanical stress-induced cell apoptosis in osteoblasts pretreated with or without specific inhibitors. Osteoblasts were pretreated with or without specific inhibitors for 1 h and then treated with mechanical stress of 0.6Hz×8cm (0 as control) for 30min and then cultured for a further 24h. (A) Apoptosis assayed by flow cytometry using annexin-V/PI double staining. (B) Quantitative analysis of apoptotic osteoblasts in each group. *P<0.05 versus control group; #*P*<0.05 versus loaded group. Data are presented as mean ± SD of three independent experiments.

Firstly, we pretreated the cells with the ERK MAPK inhibitor, PD98059, JNK inhibitor, SP600125, p38 MAPK inhibitor, SB203580, or PI3K/Akt inhibitor, LY294002. Then the cells were subjected to mechanical loading of 0.6Hz×8cm for 30 min to clarify whether the inhibitors had any effect on mechanical stimulation-induced phosphoralation changes of MAPKs and PI3K/Akt pathways. The results demonstrated that mechanical stimulation increased the phosphorylation of ERK, JNK, p38 and Akt compared with control cells (*P* < 0.05), however, the inhibitors partially blocked this effect (*P* < 0.05) (Fig 4). Secondly, in order to clarify which pathway mediated the apoptotic effect of mechanical stress, we explored the effect of specific inhibitors on the mechanical stimulation-induced osteoblasts apoptosis. Cell apoptosis was determined by using Annexin V-FITC/PI flow cytometric analysis. The results showed that mechanical loading of 0.6Hz×8cm significantly induced apoptosis compared with control cells (*P* < 0.05). Inhibition of the PI3K/Akt pathway by LY294002 caused a significant increase in mechanical stimulation-induced apoptosis compared with the mechanical loading group (*P* < 0.05). In addition, JNK inhibitor, SP600125 partly eliminated the apoptotic effect of mechanical stimulation on cell apoptosis (*P* < 0.05). However, treatment with inhibitors alone had no significant effect on the apoptosis of osteoblasts (Fig 5). The results indicated that activation of PI3K/Akt signaling pathway exhibited anti-apoptotic properties and JNK signaling pathway was closely associated with inducing of apoptosis.

### Large-magnitude mechanical stress induced osteoblasts apoptosis by regulating Bad, Bcl-2, Bax and activation of caspases-3

**Fig 6:**
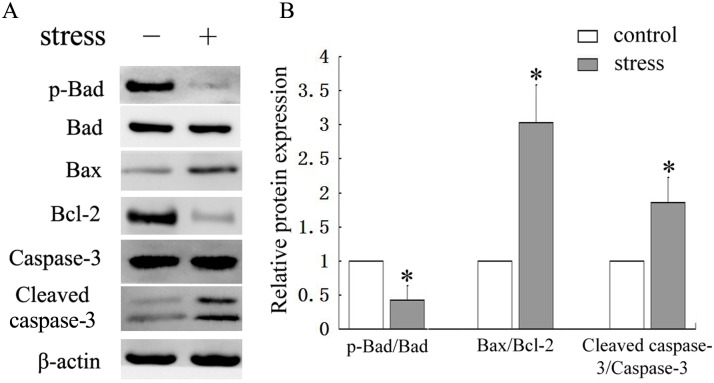
Effect of large-magnitude mechanical stress on apoptosis-related proteins expression in osteoblasts. Osteoblasts were treated with large-magnitude mechanical stress (0.6Hz×8cm) for 30min and then cultured for a further 24h. (A) Cell lysates were analyzed by western blot. (B) Quantitative analysis of expression of p-Bad, Bax, Bcl-2 and caspase-3. *P<0.05 versus control group. Each value is presented as mean ± SD of three independent experiments and data of the treatment group was expressed as fold change vs. that of control group (labeled as “1.00”) after normalized to β-actin.

To clarify the possible mechanisms by which large-magnitude mechanical stress induces osteoblasts apoptosis, western blot analysis was performed to examine apoptosis-associated proteins, including Bad, Bcl-2, Bax and caspase-3. The cells were treated with mechanical stress of 0.6Hz×8cm for 30min and then cultured for another 24h, following which the proteins were examined. The results demonstrated that large-magnitude mechanical stress increased the protein expression of Bax and caspase-3, and decreased the protein expression of Bcl-2 and p-Bad in the osteoblasts compared with the untreated cells (*P* < 0.05) (Fig 6).

### JNK and Akt activated by large-magnitude mechanical stress negatively regulated osteoblasts apoptosis

**Fig 7:**
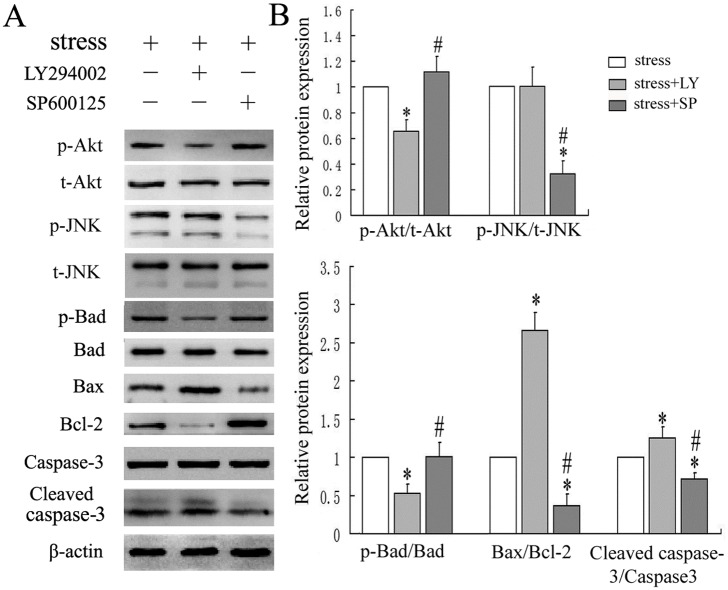
Apoptosis-related proteins expression in osteoblasts pretreated with or without JNK and Akt inhibitors respectively. Osteoblasts were pretreated with or without specific inhibitors for 1 h and then treated with mechanical stress of 0.6Hz×8cm (0 as control) for 30min and then cultured for a further 24h. (A) Cell lysates were analyzed by western blot. (B) Quantitative analysis of expression of p-Bad, Bax, Bcl-2 and caspase-3. *P<0.05 versus stress group; #*P*<0.05 versus stress+LY294002 group. Each value is presented as mean ± SD of three independent experiments and data of the treatment group was expressed as fold change vs. that of control group (labeled as “1.00”) after normalized to β-actin.

**Fig 8:**
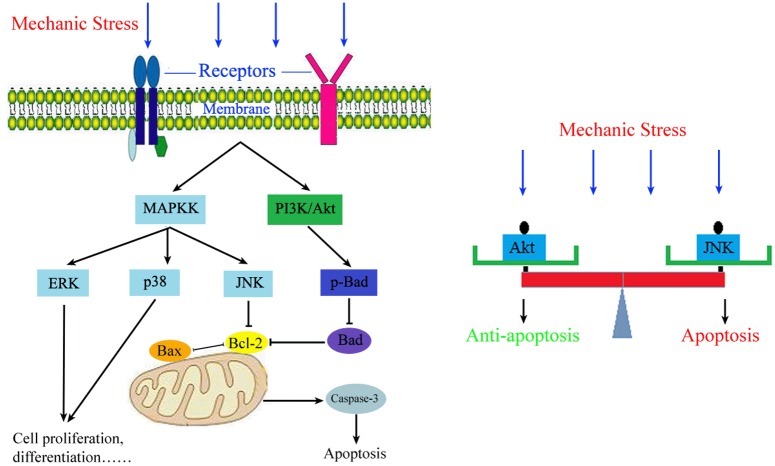
Signaling pathways in regulating the osteoblasts apoptosis. The fine balance of apoptotic and anti-apoptotic effect of mechanical stress on osteoblasts is achieved by the actions of critical signaling pathways and key signaling proteins such as JNK and Akt.

To further determine the role of JNK and Akt activation in mechanical load-induced osteoblasts apoptosis, western blot was performed to examine the apoptosis-associated proteins after cells were pretreated with the JNK or PI3K/Akt inhibitor and then subjected to mechanical loading of 0.6Hz×8cm for 30 min followed by cultured for another 24h. The results revealed that blocking JNK activity with SP600125 could up-regulate the expression of Bcl-2 and down-regulate the expression of Bax and caspase-3(*P* < 0.05) but had no significant effect on Bad phosphorylation under mechanical stress. However, PI3K/Akt inhibitor, LY294002 partly blocked the anti-apoptotic properties through up-regulating the expression of Bax and caspase-3 and down-regulating the expression of Bcl-2 and p-Bad (*P* < 0.05). In addition, inhibition of JNK or PI3K/Akt did not lead to changes of each other (Fig 7). Results from this part of experiment indicated that activation of JNK and Akt by mechanical stress independently resulted in an opposite effect on cell apoptosis via a common Bcl/Bax/caspase-3 apoptotic pathway. The difference was that Bad phosphorylation was not involved in JNK mediated pro-apoptotic effect (Fig 8).

## Discussion

To the best of our knowledge, our results have demonstrated for the first time the apoptotic effects of mechanical stress generated by liquid drops on monolayer osteoblasts. As this method had never been reported, there were no literature reports to guide us regarding the frequency and magnitude of the applied stress. Therefore, we divided osteoblasts into many experimental groups according to different levels of stress and stress duration in order to detect the apoptotic effect of mechanical stress. For the present report, we have chosen to show the most representative groups. Because the distribution, magnitude and change in stress loads on the monolayer cells were too complex to measure or calculate, the values of all stress levels in each group are represented as frequency (Hz) × height (cm).

In the present study, we found an interesting trend in apoptosis by using quantitative TUNEL analysis and caspase-3 activity assays to determine the effects of different levels of mechanical stress on osteoblasts. Our results showed that small-magnitude mechanical stress exerted anti-apoptotic effects on osteoblasts, while large-magnitude mechanical stress reversed this effect and promoted apoptosis. Mechanical stress has been reported to suppress apoptosis in osteoblasts[1, 6, 14, 32], in agreement with our observations, we also found that small-magnitude mechanical stress-treated osteoblasts showed an anti-apoptotic trend. However, as only a relatively small proportion of osteoblasts undergo apoptosis naturally, the difference between stress-treated groups and the control group was not significant by TUNEL analysis. However, results of caspase-3 activity assay demonstrated a significant decrease in the cellular apoptotic rate compared with control cells. Intriguingly, as mechanical stress increased, the number of apoptotic osteoblasts also increased. We propose that combining TUNEL and caspase-3 assays may produce a more reliable result than TUNEL alone to show that the apoptotic effect of mechanical stress in osteoblasts is dose-dependent.

There are a number of signaling pathways that can induce apoptosis. Since it has been demonstrated that MAPKs and PI3K/Akt signaling pathways can mediate cell survival[29, 33–36], we examined changes in signaling proteins in these two pathways. Although both signaling pathways were activated by small-magnitude mechanical stress, phosphorylation of Akt and ERK significantly decreased, while phosphorylation of JNK and p38 MAPK increased when large-magnitude mechanical stress was loaded. To explore the relationship between the apoptotic effect of mechanical stress and activation of signaling pathways, we used specific inhibitors to clarify which pathway mediated the apoptotic effect on osteoblasts. We observed that inhibiting the kinase activity of Akt led to an enhanced apoptotic effect, while inhibition of JNK resulted in an anti-apoptotic effect. However, inhibiting the kinase activity of other MAPKs such as p38 and ERK had no effect on apoptosis. These results suggested that JNK and Akt activation participated in the regulation of osteoblast apoptosis when mechanical stress was loaded on the cells.

Activation of JNK by large-magnitude mechanical stretch has been reported to result in cellular apoptosis[15]. It was also demonstrated that activation of the PI3K/Akt signaling pathway rescued osteoblasts from TNF-α-induced apoptosis and that inhibition of this pathway partially blocked the anti-apoptotic effect[1, 32]. Although different methods of mechanical stimulation were used, the results of these two studies are consistent with our observations. It is widely accepted that the PI3K/Akt signaling pathway mediates the anti-apoptotic effect, but the roles of the three MAPK pathways in mechanical stress-induced apoptosis are still not clear. It was suggested that mechanical stress-induced apoptosis in chondrocytes was mediated by all three MAPK pathways, while another study reported that activation of ERK produced anti-apoptotic effects in cyclic stretch-induced annulus fibrosus cells apoptosis[10, 14]. Our results indicated that p38 and ERK were not involved in osteoblast apoptosis induced by mechanical stress, in contrast to these two reports. These discrepancies may be related to differences in the cell types cultured and methods of mechanical stimulation. Further research will be required to determine the precise molecular mechanism.

The caspase family pathway is considered to be of great importance in apoptosis, as many apoptotic signaling pathways ultimately activate caspase cascades[16]. Activated caspases initiate protein degradation and are responsible for cell apoptosis[37]. Of the many signaling pathways to activate the caspase family, the Bcl-2 family plays an important role in many cell types. Bcl family proteins can be divided into anti-apoptotic proteins (Bcl-2, Bcl-xL, Bcl-w and Mcl-1) and pro-apoptotic proteins (Bax, Bad, Bak, Bik and Bid)[17]. Bax promotes apoptosis by the release of cytochrome c from mitochondria and downstream activation of caspases, while Bcl-2 inhibits cell apoptosis by restraining the pro-apoptotic effects of Bax and blocking the release of cytochrome c[38, 39]. Therefore, the Bax/Bcl-2 ratio is important for controlling cell apoptosis by regulating activation of effective cleaved caspases. Phosphorylated Bad inhibits cell apoptosis by dissociating Bcl-2 and Bcl-XL from Bcl-XL-Bad and Bcl-2-Bad heterodimers, respectively, which leads to a decreased Bax/Bcl-2 ratio[40]. We found that large-magnitude mechanical stress resulted in increased expression of Bax protein and decreased expression of p-Bad and Bcl-2. Caspase-3 is considered to be a central mediator of apoptosis and is one of the major activated cysteine proteases in the caspase family that is pivotal in apoptosis[41]. It was reported that cyclic stretch-induced apoptosis was controlled by caspase-3[42]. Considering its importance in apoptosis, we observed cleaved caspase-3 protein levels in the current study, indicating an association with increased apoptosis in osteoblasts responding to large-magnitude mechanical stress. Our results thus indicate that large-magnitude mechanical stress may induce cell apoptosis by the Bax/Bcl-2/caspase-3 pathway. By using specific inhibitors, we found that inactivation of the PI3K/Akt pathway partially blocked the anti-apoptotic effect in osteoblasts by down-regulating p-Bad, which resulted in an increased Bax/Bcl-2 ratio and activation of caspase-3. However, the JNK pathway exerted a pro-apoptotic effect by directly increasing the Bax/Bcl-2 ratio and not by altering levels of the pro-apoptotic protein Bad. Since the interaction of these intracellular signaling pathways form a complex network, we also examined the effect of inhibiting one signaling pathway on the other. Our results showed that phosphorylation of JNK and Akt by mechanical stress were independent.

The present study demonstrates that mechanical stress induces apoptosis in osteoblasts by activating the JNK pathway and inhibiting the activation of the PI3K/Akt pathway. As activation of JNK leads to apoptosis, while Akt protects against it, we propose that the regulation of mechanical stress on osteoblast apoptosis is controlled by a balance of effects (Fig 8). Mechanical stimulation can induce phosphorylation of JNK and Akt simultaneously, but the PI3K/Akt signaling pathway is significantly activated only under small-magnitude mechanical stress to produce an anti-apoptotic effect. As the mechanical load increases, phosphorylation of JNK increases and phosphorylation of Akt is decreased, thus inducing an apoptotic effect. However, we did not investigate the mechanisms underlying the different levels of JNK and PI3K/Akt activation produced by varying levels of mechanical stress or other signaling pathways involved in the apoptotic effect. Although our understanding of mechanical stress-mediated apoptotic effects in osteoblasts remains poor and requires further investigation, our findings do provide a new model to simulate the stress environment of osteoblasts and offer as a complementing for the mechanisms in the regulation of mechanical stress-mediated apoptosis in osteoblasts.

In summary, the above results increase our understanding of the mechanisms associated with osteoblast apoptosis in response to mechanical forces, which is partly mediated through JNK MAPK and PI3K/Akt pathway regulation of Bax/Bcl-2/caspase-3 activation, resulting in inhibition of apoptosis upon small-magnitude stress and increased apoptosis upon large-magnitude stress. Further work will be necessary to investigate the precise molecular mechanisms regulating osteoblast apoptosis, proliferation and differentiation in response to mechanical stress.

## References

[1] Pavalko FM, Gerard RL, Ponik SM, Gallagher PJ, Jin Y, Norvell SM. Fluid shear stress inhibits TNF-alpha-induced apoptosis in osteoblasts: a role for fluid shear stress-induced activation of PI3-kinase and inhibition of caspase-3. Journal of cellular physiology. 2003;194(2):194–205. 10.1002/jcp.10221 . 12494458

[2] Aisha MD, Nor-Ashikin MN, Sharaniza AB, Nawawi H, Froemming GR. Orbital fluid shear stress promotes osteoblast metabolism, proliferation and alkaline phosphates activity in vitro. Experimental cell research. 2015;337(1):87–93. 10.1016/j.yexcr.2015.07.002 . 26163894

[3] Ozcivici E, Luu YK, Adler B, Qin YX, Rubin J, Judex S, et al Mechanical signals as anabolic agents in bone. Nat Rev Rheumatol. 2010;6(1):50–9. 10.1038/nrrheum.2009.239. WOS:000273247300009. 20046206 PMC3743048

[4] Bucaro MA, Fertala J, Adams CS, Steinbeck M, Ayyaswamy P, Mukundakrishnan K, Shapiro IM, et al Bone cell survival in microgravity: evidence that modeled microgravity increases osteoblast sensitivity to apoptogens. Ann N Y Acad Sci. 2004;1027:64–73. 10.1196/annals.1324.007 15644346

[5] Hillsley MV, Frangos JA. Bone tissue engineering: the role of interstitial fluid flow. Biotechnology and bioengineering. 1994;43(7):573–81. 10.1002/bit.260430706 . 11540959

[6] Bin G, Bo Z, Jing W, Jin J, Xiaoyi T, Cong C, et al Fluid shear stress suppresses TNF-alpha-induced apoptosis in MC3T3-E1 cells: Involvement of ERK5-AKT-FoxO3a-Bim/FasL signaling pathways. Experimental cell research. 2016;343(2):208–17. 10.1016/j.yexcr.2016.03.014 . 27060196

[7] Cheng WP, Wang BW, Lo HM, Shyu KG. Mechanical Stretch Induces Apoptosis Regulator TRB3 in Cultured Cardiomyocytes and Volume-Overloaded Heart. PloS one. 2015;10(4):e0123235 10.1371/journal.pone.0123235 25898323 PMC4405267

[8] Yanagisawa M, Suzuki N, Mitsui N, Koyama Y, Otsuka K, Shimizu N. Compressive force stimulates the expression of osteogenesis-related transcription factors in ROS 17/2.8 cells. Archives of oral biology. 2008;53(3):214–9. 10.1016/j.archoralbio.2007.08.012 . 18054892

[9] Song H, Liang W, Xu S, Li Z, Chen Z, Cui W, et al A novel role for integrin-linked kinase in periodic mechanical stress-mediated ERK1/2 mitogenic signaling in rat chondrocytes. Cell biology international. 2016;40(7):832–9. 10.1002/cbin.10622 . 27154044

[10] Kong DC, Zheng TS, Zhang M, Wang DD, Du SH, Li X, et al Static Mechanical Stress Induces Apoptosis in Rat Endplate Chondrocytes through MAPK and Mitochondria-Dependent Caspase Activation Signaling Pathways. PloS one. 2013;8(7):e69403 ARTN e69403 10.1371/journal.pone.0069403. WOS:000322391400058. 23894471 PMC3716647

[11] Vazquez M, Evans BAJ, Riccardi D, Evans SL, Ralphs JR, Dillingham CM, et al A new method to investigate how mechanical loading of osteocytes controls osteoblasts. Front Endocrinol. 2014;5:208 Artn 208 10.3389/Fendo.2014.00208. WOS:000209749800206. 25538684 PMC4260042

[12] Bakker AD, Gakes T, Hogervorst JM, de Wit GM, Klein-Nulend J, Jaspers RT. Mechanical Stimulation and IGF-1 Enhance mRNA Translation Rate in Osteoblasts Via Activation of the AKT-mTOR Pathway. Journal of cellular physiology. 2016;231(6):1283–90. 10.1002/jcp.25228 . 26505782

[13] Iura A, McNerny EG, Zhang YS, Kamiya N, Tantillo M, Lynch M, et al Mechanical Loading Synergistically Increases Trabecular Bone Volume and Improves Mechanical Properties in the Mouse when BMP Signaling Is Specifically Ablated in Osteoblasts. PloS one. 2015;10(10):e0141345 ARTN e0141345 10.1371/journal.pone.0141345. WOS:000363248400121. 26489086 PMC4619208

[14] Zhang K, Ding W, Sun W, Sun XJ, Xie YZ, Zhao CQ, et al Beta1 integrin inhibits apoptosis induced by cyclic stretch in annulus fibrosus cells via ERK1/2 MAPK pathway. Apoptosis. 2016;21(1):13–24. 10.1007/s10495-015-1180-7 . 26467923

[15] Matsui H, Fukuno N, Kanda Y, Kantoh Y, Chida T, Nagaura Y, et al The expression of Fn14 via mechanical stress-activated JNK contributes to apoptosis induction in osteoblasts. The Journal of biological chemistry. 2014;289(10):6438–50. 10.1074/jbc.M113.536300 24446436 PMC3945310

[16] Wang L, Pan J, Wang T, Song M, Chen W. Pathological cyclic strain-induced apoptosis in human periodontal ligament cells through the RhoGDIalpha/caspase-3/PARP pathway. PloS one. 2013;8(10):e75973 10.1371/journal.pone.0075973 24130754 PMC3794943

[17] Koff JL, Ramachandiran S, Bernal-Mizrachi L. A time to kill: targeting apoptosis in cancer. International journal of molecular sciences. 2015;16(2):2942–55. 10.3390/ijms16022942 25636036 PMC4346874

[18] Jilka RL, Weinstein RS, Bellido T, Parfitt AM, Manolagas SC. Osteoblast programmed cell death (apoptosis): modulation by growth factors and cytokines. Journal of bone and mineral research: the official journal of the American Society for Bone and Mineral Research. 1998;13(5):793–802. 10.1359/jbmr.1998.13.5.793 . 9610743

[19] Plotkin LI, Gortazar AR, Davis HM, Condon KW, Gabilondo H, Maycas M, et al Inhibition of Osteocyte Apoptosis Prevents the Increase in Osteocytic Receptor Activator of Nuclear Factor kappa B Ligand (RANKL) but Does Not Stop Bone Resorption or the Loss of Bone Induced by Unloading. Journal Of Biological Chemistry. 2015;290(31):18934–42. 10.1074/jbc.M115.642090. WOS:000358781100006. 26085098 PMC4521013

[20] Jilka RL, O'Brien CA, Roberson PK, Bonewald LF, Weinstein RS, Manolagas SC. Dysapoptosis of osteoblasts and osteocytes increases cancellous bone formation but exaggerates cortical porosity with age. Journal of bone and mineral research: the official journal of the American Society for Bone and Mineral Research. 2014;29(1):103–17. 10.1002/jbmr.2007 23761243 PMC3823639

[21] Srivastava K, Tyagi AM, Khan K, Dixit M, Lahiri S, Kumar A, et al Isoformononetin, a methoxydaidzein present in medicinal plants, reverses bone loss in osteopenic rats and exerts bone anabolic action by preventing osteoblast apoptosis. Phytomedicine: international journal of phytotherapy and phytopharmacology. 2013;20(6):470–80. 10.1016/j.phymed.2012.12.021 . 23395215

[22] Johnson GL, Lapadat R. Mitogen-activated protein kinase pathways mediated by ERK, JNK, and p38 protein kinases. Science. 2002;298(5600):1911–2. 10.1126/science.1072682 . 12471242

[23] Zhang L, Zhou FF, ten Dijke P. Signaling interplay between transforming growth factor-beta receptor and PI3K/AKT pathways in cancer. Trends Biochem Sci. 2013;38(12):612–20. 10.1016/j.tibs.2013.10.001. WOS:000328590500004. 24239264

[24] Takeda K, Naguro I, Nishitoh H, Matsuzawa A, Ichijo H. Apoptosis Signaling Kinases: From Stress Response to Health Outcomes. Antioxidants & redox signaling. 2011;15(3):719–61. 10.1089/ars.2010.3392 . 20969480

[25] Nithianandarajah-Jones GN, Wilm B, Goldring CE, Muller J, Cross MJ. ERK5: structure, regulation and function. Cellular signalling. 2012;24(11):2187–96. 10.1016/j.cellsig.2012.07.007 . 22800864

[26] Giancotti FG, Ruoslahti E. Integrin signaling. Science. 1999;285(5430):1028–32. 10446041 10.1126/science.285.5430.1028

[27] Tahimic CG, Long RK, Kubota T, Sun MY, Elalieh H, Fong C, et al Regulation of Ligand and Shear Stress-induced Insulin-like Growth Factor 1 (IGF1) Signaling by the Integrin Pathway. The Journal of biological chemistry. 2016;291(15):8140–9. 10.1074/jbc.M115.693598 26865633 PMC4825016

[28] Shoham N, Gottlieb R, Sharabani-Yosef O, Zaretsky U, Benayahu D, Gefen A. Static mechanical stretching accelerates lipid production in 3T3-L1 adipocytes by activating the MEK signaling pathway. American journal of physiology Cell physiology. 2012;302(2):C429–41. 10.1152/ajpcell.00167.2011 . 22012328

[29] Davis WJ, Lehmann PZ, Li W. Nuclear PI3K signaling in cell growth and tumorigenesis. Frontiers in cell and developmental biology. 2015;3:24 10.3389/fcell.2015.00024 25918701 PMC4394695

[30] Zheng D, Zhu G, Liao S, Yi W, Luo G, He J, et al Dysregulation of the PI3K/Akt signaling pathway affects cell cycle and apoptosis of side population cells in nasopharyngeal carcinoma. Oncology letters. 2015;10(1):182–8. 10.3892/ol.2015.3218 26170996 PMC4487176

[31] Datta SR, Dudek H, Tao X, Masters S, Fu H, Gotoh Y, et al Akt phosphorylation of BAD couples survival signals to the cell-intrinsic death machinery. Cell. 1997;91(2):231–41. . 9346240 10.1016/s0092-8674(00)80405-5

[32] Bin G, Cuifang W, Bo Z, Jing W, Jin J, Xiaoyi T, et al Fluid shear stress inhibits TNF-alpha-induced osteoblast apoptosis via ERK5 signaling pathway. Biochemical and biophysical research communications. 2015;466(1):117–23. 10.1016/j.bbrc.2015.08.117 . 26325467

[33] Chuang WL, Lin PY, Lin HC, Chen YL. The Apoptotic Effect of Ursolic Acid on SK-Hep-1 Cells is Regulated by the PI3K/Akt, p38 and JNK MAPK Signaling Pathways. 2016;21(4). 10.3390/molecules21040460 . PMC6274268 27104510

[34] Roymans D, Slegers H. Phosphatidylinositol 3-kinases in tumor progression. European journal of biochemistry / FEBS. 2001;268(3):487–98. . 11168386 10.1046/j.1432-1327.2001.01936.x

[35] Kim EK, Choi E-J. Compromised MAPK signaling in human diseases: an update. Archives of toxicology. 2015;89(6):867–82. 10.1007/s00204-015-1472-2 . 25690731

[36] Yuan L, Wang J, Xiao H, Wu W, Wang Y, Liu X. MAPK signaling pathways regulate mitochondrial-mediated apoptosis induced by isoorientin in human hepatoblastoma cancer cells. Food and chemical toxicology: an international journal published for the British Industrial Biological Research Association. 2013;53(1873–6351 (Electronic)):62–8. 10.1016/j.fct.2012.11.048 . 23220614

[37] Green DR, Llambi F. Cell Death Signaling. Cold Spring Harb Perspect Biol. 2015;7(12):a006080 10.1101/cshperspect.a006080 26626938 PMC4665079

[38] Ackler S, Mitten MJ, Foster K, Oleksijew A, Refici M, Tahir SK, et al The Bcl-2 inhibitor ABT-263 enhances the response of multiple chemotherapeutic regimens in hematologic tumors in vivo. Cancer chemotherapy and pharmacology. 2010;66(5):869–80. 10.1007/s00280-009-1232-1 . 20099064

[39] Vaux DL, Korsmeyer SJ. Cell death in development. Cell. 1999;96(2):245–54. . 9988219 10.1016/s0092-8674(00)80564-4

[40] Yang E, Zha J, Jockel J, Boise LH, Thompson CB, Korsmeyer SJ. Bad, a heterodimeric partner for Bcl-XL and Bcl-2, displaces Bax and promotes cell death. Cell. 1995;80(2):285–91. . 7834748 10.1016/0092-8674(95)90411-5

[41] Yu D, Mu S, Zhao D, Wang G, Chen Z, Ren H, et al Puerarin attenuates glucocorticoid-induced apoptosis of hFOB1.19 cells through the JNK- and Akt-mediated mitochondrial apoptotic pathways. International journal of molecular medicine. 2015;36(2):345–54. 10.3892/ijmm.2015.2258 26101183 PMC4501663

[42] Zhong W, Xu C, Zhang F, Jiang X, Zhang X, Ye D. Cyclic stretching force-induced early apoptosis in human periodontal ligament cells. Oral diseases. 2008;14(3):270–6. 10.1111/j.1601-0825.2007.01375.x . 18208476

